# Total calcanectomy in chronic osteomyelitis of calcaneus: A case report

**DOI:** 10.1051/sicotj/2026064

**Published:** 2026-10-13

**Authors:** Kumar Shantanu Anand, Rakhee Anand, Gyan Ranjan

**Affiliations:** Anand Hospital Purnea-854301 Bihar India

**Keywords:** Chronic osteomyelitis of calcaneus, Total calcanectomy, Calcaneal osteomyelitis, Calcaneum

## Abstract

Chronic osteomyelitis of the calcaneus, although uncommon, is associated with substantial morbidity and remains one of the most challenging forms of chronic osteomyelitis to treat, causing significant patient morbidity. The condition is rather refractory to various treatment modalities and sometimes may necessitate below-knee amputation for complete eradication of infection. Total calcanectomy, in such a scenario, can be a limb salvage (foot salvage) procedure in carefully selected patients. Here we report a case of Cierny–Mader stage IV chronic osteomyelitis of the calcaneus treated by means of total calcanectomy and application of antibiotic (vancomycin and gentamicin) impregnated beads of synthetic absorbable calcium sulfate powder (STIMULAN, Biocomposites, UK), with a follow-up period of 6 years and 2 months – complete eradication of infection while preserving the weight-bearing functions of foot, has been successfully achieved. To our knowledge, this is among the first few reported cases of total calcanectomy combined with application of synthetic absorbable antibiotic-loaded calcium sulphate (STIMULAN) beads for chronic diffuse calcaneal osteomyelitis.

## Introduction

Chronic osteomyelitis of the calcaneus is uncommon (accounting for about 7–8% of all osteomyelitis cases in adults) [1] but difficult to treat because infection often involves both the bone and the surrounding soft tissues. Repeated debridement and prolonged antibiotic therapy may fail to eradicate the disease, particularly in patients with extensive calcaneal involvement, multiple discharging sinuses, and compromised soft-tissue cover. In such cases, below-knee amputation may be considered to control infection.

Total calcanectomy is a limb-salvage procedure that allows complete excision of the infected calcaneus while preserving a plantigrade, weight-bearing foot. The use of locally delivered antibiotics may further assist in managing the residual dead space and achieving a high antibiotic concentration at the site of infection.

We report a case of Cierny–Mader stage IV [2, 3] chronic calcaneal osteomyelitis successfully treated by total calcanectomy and implantation of synthetic absorbable calcium-sulphate beads (STIMULAN, Biocomposites, UK) loaded with vancomycin and gentamicin, resulting in eradication of infection and restoration of functional weight-bearing [4]. To our knowledge, this is one of the first few reported cases of total calcanectomy combined with synthetic absorbable antibiotic-loaded calcium sulphate beads for chronic diffuse calcaneal osteomyelitis.

### Case report

A 28-year-old male construction worker presented with a three-year history of pain, swelling, and persistent discharging sinuses involving the right heel. He had previously undergone surgical debridement twice at other institutions and received multiple courses of antibiotics, but continued to experience chronic pain, recurrent wound discharge, and progressive inability to bear weight on the affected heel. On examination, there were multiple active sinuses over the heel, surrounding hyperpigmented and scarred skin, wasting of the right thigh and leg muscles, and marked tenderness over the calcaneus.

Plain radiographs demonstrated extensive destruction of the calcaneus with diffuse sclerosis and multiple irregular lytic areas, consistent with advanced chronic osteomyelitis (Figure 1). Based on the clinical presentation and radiographic findings, the condition was diagnosed as Cierny–Mader stage IV chronic calcaneal osteomyelitis, indicating diffuse involvement of the entire calcaneus.

**Figure 1:**
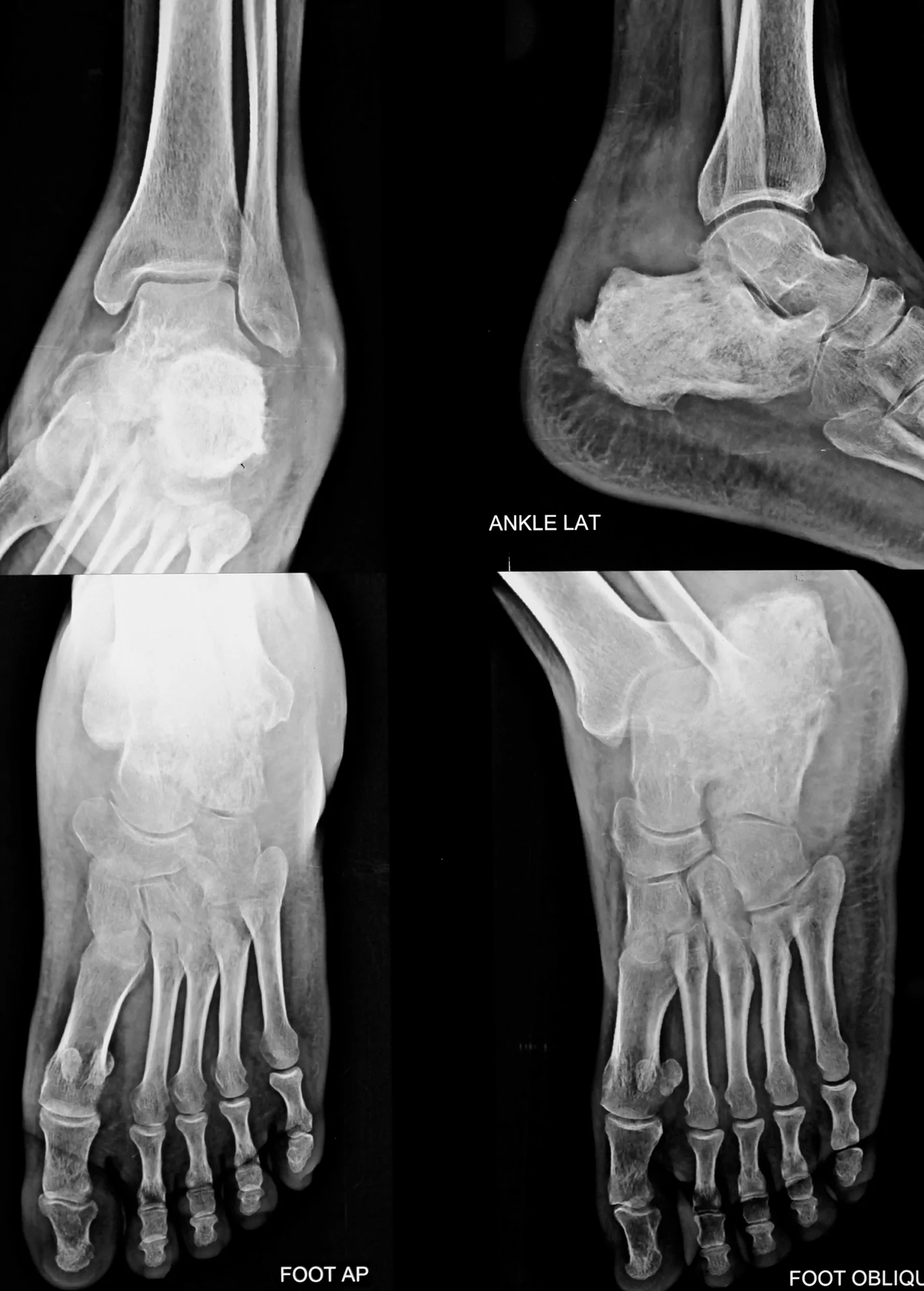
Pre-operative X-ray.

The patient had persistent infection despite previous surgical treatment, extensive osseous involvement, multiple draining sinuses, significant functional impairment, and poor prospects for successful limb salvage with further debridement alone. Although the patient requested a below-knee amputation because of prolonged disability, a limb-salvage procedure was considered feasible. After detailed counselling regarding the available treatment options, including the potential risks and benefits of amputation versus limb salvage, total calcanectomy was selected with the objectives of complete eradication of infection while preserving a painless, plantigrade, weight-bearing foot [5].

The procedure was performed through a modified Gaenslen approach [6–10]. The incision extended proximally about seven centimeters above the heel vertically (same as the vertical incision of extensile lateral approach) and was continued inferomedially around the sinus tracts and ended just distal to the calcaneocuboid joint (Figure 2). The incision was deepened to the subperiosteal plane and longitudinal in order to maintain the continuity of the tendocalcaneus with the plantar fascia. The calcaneus was dissected out along with the infected granulation tissue. Copious irrigation with normal saline, followed by povidone-iodine and hydrogen peroxide lavage, was performed and hemostasis was achieved. The heel fat pad was meticulously preserved throughout. The dead space created after excision of the calcaneus was filled with antibiotic (vancomycin and gentamicin) impregnated beads of synthetic absorbable calcium sulfate powder (STIMULAN, Biocomposites, UK) – these beads were prepared at the time of surgery. The incision was closed over a thin corrugated drain, and the limb was placed over a below-knee splint.

**Figure 2:**
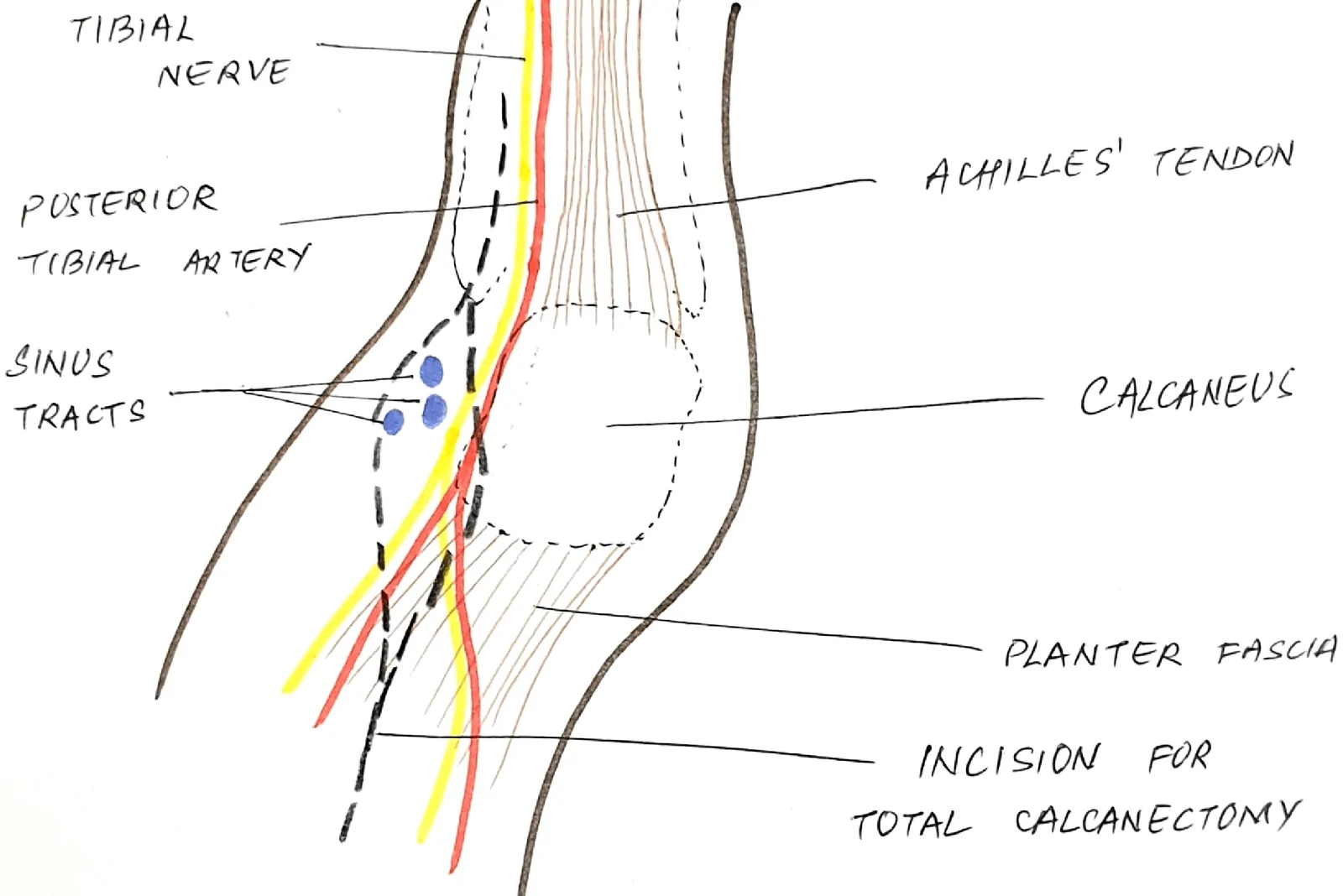
Incision diagram drawn by the author.

Postoperatively, the patient received intravenous ceftriaxone for seven days followed by three weeks of oral co-amoxiclav. Partial weight bearing was commenced at three weeks and full weight bearing at six weeks. The patient was followed up at regular intervals for his clinical and radiological assessment of recovery. X-rays done postoperatively at 13 months (Figure 3) and 25 months (Figure 4) are reproduced here. The patient had returned to full-time work three months after surgery and has remained independently ambulatory with a painless, plantigrade foot. At the latest follow-up of 6 years and two months, there was no clinical or radiographic evidence of recurrent infection (Figures 5a, 5b).

**Figure 3:**
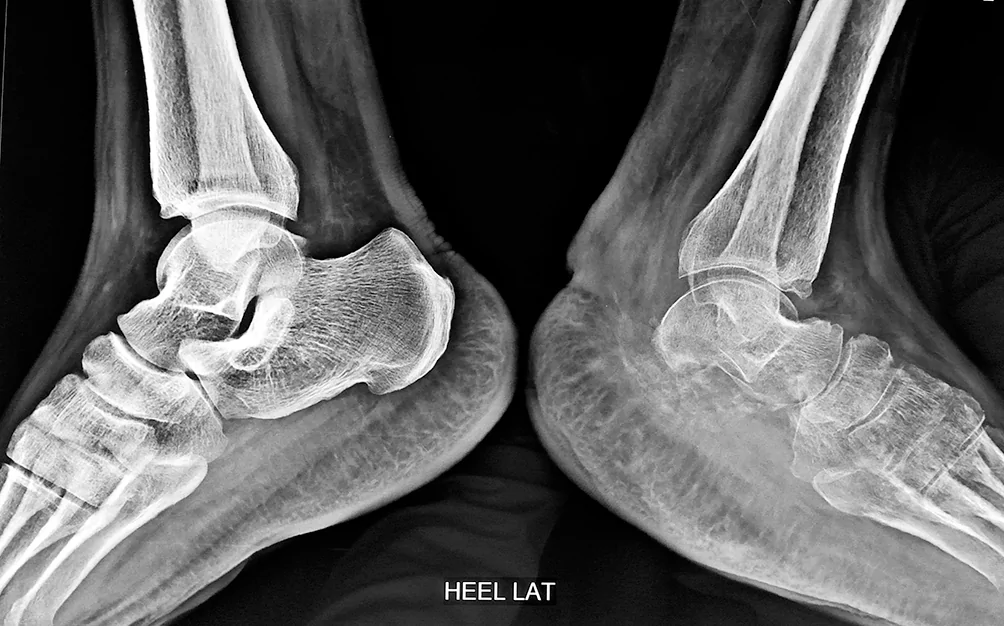
Post-operative X-ray at 13 months.

**Figure 4:**
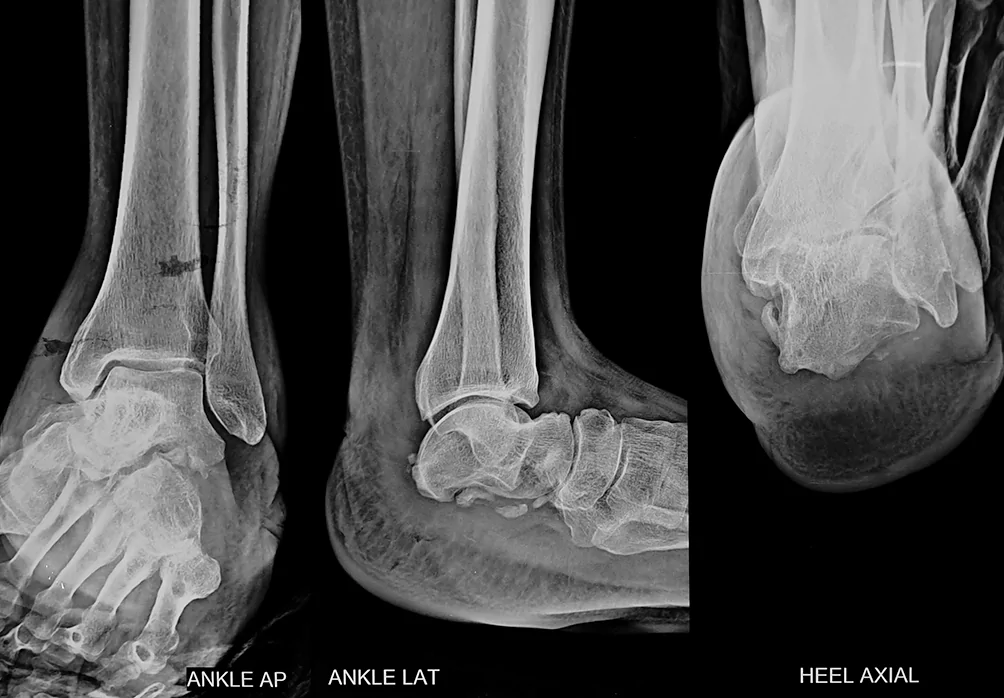
Post-operative X-ray at 25 Months.

**Figure 5:**
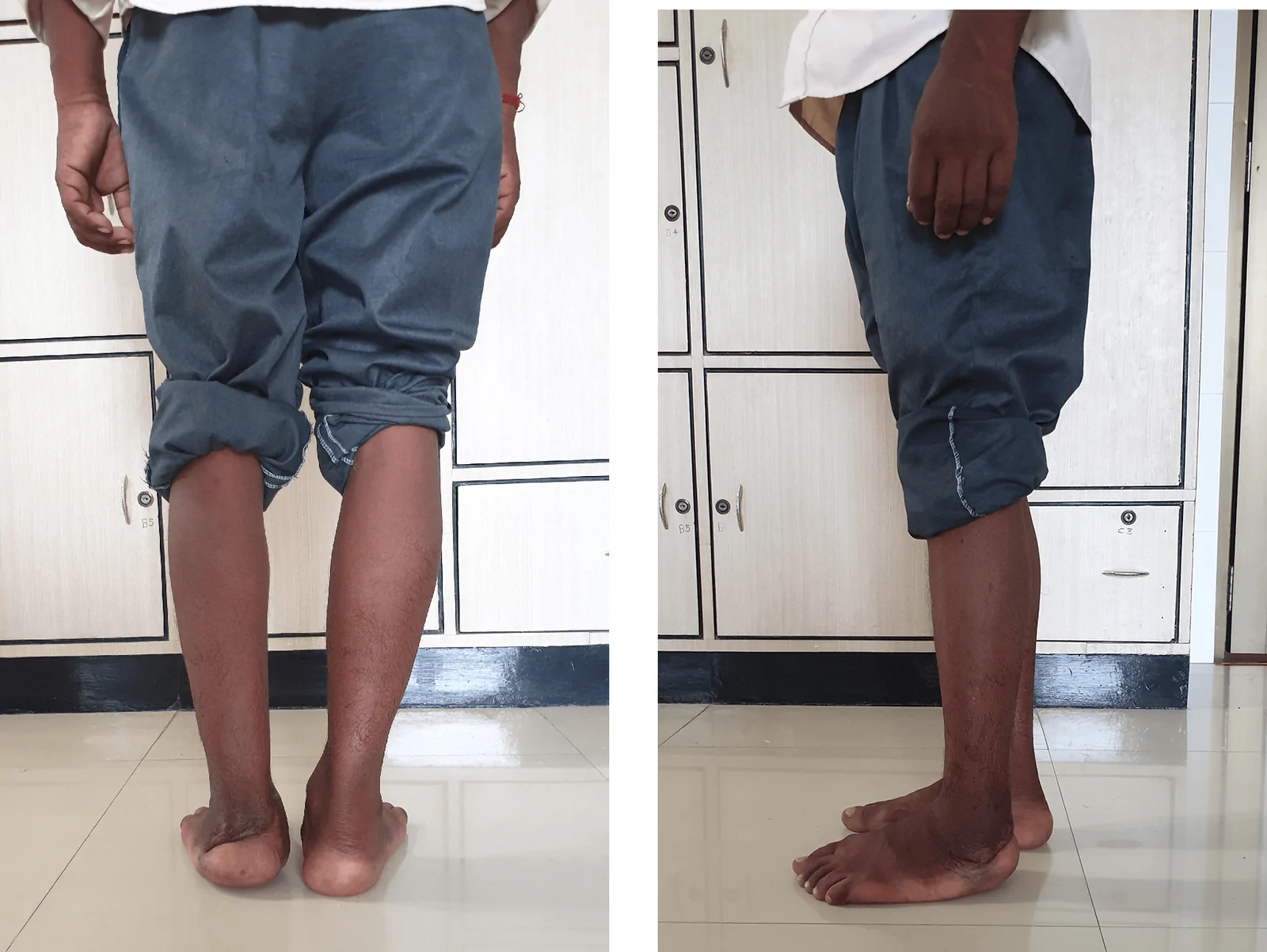
(A) Post op picture (Standing, back) at 6 years. (B). Post op picture (Standing, side) at 6 years.

## Discussion

**1. Background and summary of the present case:** Chronic calcaneal osteomyelitis is an uncommon but particularly challenging form of osteomyelitis because the calcaneus is a weight-bearing bone with limited soft-tissue coverage and relatively poor vascularity. Persistent infection frequently results in chronic pain, recurrent sinus formation, impaired ambulation, and repeated surgical procedures. In the present case, a young manual labourer with Cierny–Mader stage IV chronic calcaneal osteomyelitis failed multiple previous operations before being successfully treated with total calcanectomy combined with local antibiotic delivery using absorbable calcium sulphate beads. At 6 years and two months of follow-up, infection had been totally eradicated, the patient had returned to full-time work, and a painless, plantigrade, weight-bearing foot was preserved.

**2. Limitations:** This report has several limitations. Although this report describes a single patient with a long follow-up of 6 years and 2 months, it cannot establish the superiority of total calcanectomy over other treatment strategies, as we understand a larger patient series is desirable. Functional outcome was assessed clinically without standardized patient-reported outcome measures, and conclusions regarding long-term durability should be interpreted cautiously. Nevertheless, chronic diffuse calcaneal osteomyelitis requiring total calcanectomy is uncommon, and detailed reports remain limited in the literature. This case, therefore, contributes additional evidence regarding limb-salvage surgery in this difficult clinical setting.

**3. Challenges in chronic calcaneal osteomyelitis:** The management of chronic calcaneal osteomyelitis remains difficult because infection usually involves not only the calcaneus but also the surrounding soft tissues, producing extensive fibrosis, multiple discharging sinuses, dead space after debridement, and compromised local vascularity [9, 10]. Repeated debridement and prolonged systemic antibiotic therapy often fail to eradicate infection completely, particularly in diffuse (Cierny–Mader stage IV) disease. Consequently, patients frequently undergo multiple procedures before definitive treatment is achieved, and below-knee amputation has historically been considered the definitive solution for persistent infection. Previous series have reported that complete eradication of calcaneal osteomyelitis remains difficult despite aggressive treatment, highlighting the need for reliable limb-salvage strategies.

**4. Why total calcanectomy:** Total calcanectomy aims to achieve complete excision of infected bone while preserving the heel pad, Achilles tendon continuity, and a stable, plantigrade foot. Compared with repeated limited debridement, complete removal of the infected calcaneus eliminates the principal nidus of infection and creates a more favourable biological environment for healing. Earlier reports by Martini and colleagues [9] and Baumhauer et al. demonstrated encouraging rates of infection eradication following total calcanectomy, although relatively few contemporary reports have described this procedure. Our experience supports these observations and suggests that total calcanectomy remains a valuable limb-salvage option in carefully selected patients with diffuse calcaneal involvement.

**5. Role of local antibiotic delivery:** An important feature of the present case was the adjunctive use of synthetic absorbable calcium sulphate (STIMULAN) as a local antibiotic delivery system. Recent systematic reviews have reported infection eradication rates approaching 90–92% when antibiotic-loaded calcium sulphate is used as an adjunct to meticulous surgical debridement for chronic osteomyelitis [11]. Following radical debridement and total calcanectomy, the dead space was filled with calcium sulphate beads impregnated with vancomycin and gentamicin, allowing sustained local release of high antibiotic concentrations directly into the surgical site while minimizing systemic toxicity. In addition to providing effective dead-space management, the absorbable nature of the material eliminates the need for a second procedure for removal, unlike polymethylmethacrylate (PMMA) antibiotic cement. Although local antibiotic carriers have been described in the management of chronic osteomyelitis, their use in conjunction with total calcanectomy for diffuse chronic calcaneal osteomyelitis has been infrequently reported. In our patient, this strategy may have contributed to successful infection eradication and uneventful wound healing, although the favourable outcome should be attributed to the combination of meticulous surgical debridement, complete excision of infected bone, culture-directed systemic antibiotics, and structured rehabilitation rather than the local antibiotic carrier alone.

**6. Lessons from tumour surgery and complex reconstruction:** The principles of total calcanectomy are comparable to those employed in musculoskeletal tumour surgery, where complete excision of diseased bone is frequently preferred over repeated incomplete procedures. Following calcanectomy, numerous reconstructive techniques have been described, including vascularized bone grafts, custom prostheses, and allograft reconstruction. However, these procedures are technically demanding, increase operative time, and may themselves introduce additional complications. In contrast, preservation of the heel pad combined with gradual functional rehabilitation often provides satisfactory walking ability without complex reconstruction. The favourable functional recovery in our patient supports the concept that, in selected cases, a simple and biologically sound reconstruction may achieve excellent functional results while minimizing surgical morbidity.

**7. Clinical implications:** This case highlights that total calcanectomy should not be regarded solely as a salvage procedure immediately preceding amputation. In appropriately selected patients with diffuse chronic calcaneal osteomyelitis, preservation of the heel pad together with complete excision of infected bone and local antibiotic therapy may eradicate infection while maintaining independent ambulation and return to work. Careful patient selection, meticulous surgical technique, and thorough postoperative rehabilitation remain essential for successful outcomes. To our knowledge, this is one of the first few published reports describing total calcanectomy combined with absorbable antibiotic-loaded calcium sulphate (STIMULAN) for diffuse chronic calcaneal osteomyelitis, thereby expanding the available evidence on adjunctive local antibiotic therapy in limb-salvage surgery.

## Conclusion

Although further studies with larger cohorts are required, this case adds to the limited body of evidence supporting total calcanectomy as a reliable limb-salvage procedure for advanced chronic calcaneal osteomyelitis. When combined with effective local antibiotic delivery, it can achieve infection eradication while preserving a functional, weight-bearing foot (Table 1). This case, which, to our knowledge, is among the first few reported cases of total calcanectomy combined with application of synthetic absorbable antibiotic-loaded (vancomycin and gentamicin) calcium sulphate (STIMULAN) beads for chronic diffuse calcaneal osteomyelitis, also demonstrates the potential value of adjunctive local antibiotic delivery using absorbable calcium sulphate beads loaded with vancomycin and gentamicin following total calcanectomy. Although larger studies are required to confirm its clinical benefit, this technique offers a promising strategy for dead-space management and local infection control in chronic calcaneal osteomyelitis.

**Table 1:** Clinical indications, treatment, infection eradication, and functional outcomes following total calcanectomy: Comparison of published studies with the present case.

Study	Year	No. of patients	Indication	Procedure	Infection eradication	Functional outcome	Follow-up period
Baumhauer et al. [5]	1998	8	Chronic calcaneal osteomyelitis	Total calcanectomy	All patients (No need for amputation)	Ambulation preserved in most patients	27 months
Jain et al. [12]	2020	2	Neuropathic heel ulcer + osteomyelitis	Total calcanectomy	All patients (No need for amputation)	Returned to activities of daily living	18 months
Pereira et al. [13]	2022	6	Chronic calcaneal osteomyelitis	Total calcanectomy	4 out of 6 patients	Walking ability maintained in survivors	1–12 years
**Present case**	2026	1	Cierny–Mader IV chronic calcaneal osteomyelitis	Total calcanectomy + antibiotic calcium sulphate beads	Complete eradication of infection (No need for amputation)	Returned to construction work; painless plantigrade foot	6 years 2 months

## Data Availability

The data supporting the findings of this case report are included within the article. Additional anonymized clinical data are available from the corresponding author upon reasonable request, subject to institutional policies and protection of patient confidentiality.
